# Lactococcus garvieae

**DOI:** 10.3201/eid3208.260970

**Published:** 2026-08

**Authors:** Clyde Partin

**Affiliations:** Emory University School of Medicine, Atlanta, Georgia, USA

**Keywords:** Bacteria, Lactococcus garvieae, bovine mastitis, Ellen I. Garvie, National Institute for Research in Dairying, Streptococcus garvieae, etymology, food safety

## *Lactococcus garvieae* [lak″tō-kah′-kuhs gar′-vē-ә]

Microbiologist Ellen I. Garvie achieved a prolific oeuvre working at the National Institute for Research in Dairying in Shinfield, UK, during the 1960s and 1970s (Figure). Yet her biographical footprint is sparse, a commentary on society’s failure to adequately acknowledge women scientists. In 1983, Garvie was duly recognized for her accomplishments when the bacterium *Streptococcus garvieae*, a causative agent of bovine mastitis, was named in her honor. After taxonomy clarification, primarily on the basis of similarity of lipid biochemistry, *S. garvieae* was transferred to the *Lactococcus* (*lac-*, Latin for milk; -*coccus*, meaning sphere) epithet in 1985. *L. garvieae* emerged as a threat to fish and to humans consuming infected fish. In further recognition of Garvie’s work, this issue includes a case report of a serious *L. garvieae* bloodstream infection (page 1382) and a first-hand account of the patient’s harrowing encounter with the organism (page 1354).

**Figure:**
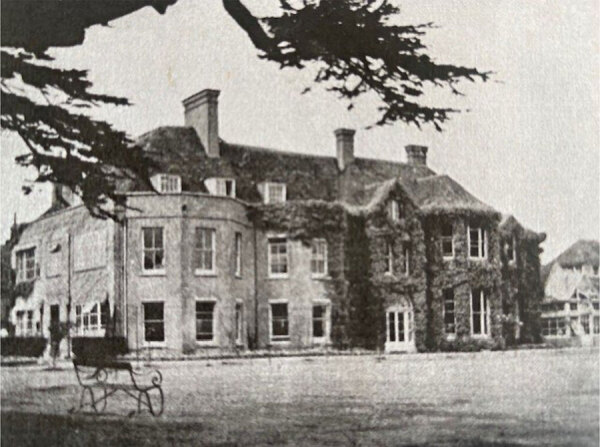
Manor House on the 350-acre Shinfield Manor estate, which later became home to the National Institute for Research in Dairying, Shinfield, England, after being founded in 1912. The Bacteriology and Metabolic Departments with associated laboratories were housed here from 1923 to 1992.  Microbiologist Ellen I. Garvie would have worked here, researching the problem of dairy product contamination. Part of the Manor House dated to the 17th century. It was demolished in 2001. Photograph published in Proceedings of the Royal Society of London, 1951.
